# Supplementary material for: Identifying and characterizing pesticide use on 9,000 fields of organic agriculture
Source: Nat Commun. 2021 Sep 15;12:5461. doi: 10.1038/s41467-021-25502-w (PMC8443594; doi:10.1038/s41467-021-25502-w)
Supplement: Supplementary file 1 — Supplementary Information [file 41467_2021_25502_MOESM1_ESM.pdf]

Identifying and characterizing pesticide use on 9,000 fields of organic agriculture.

Supplementary Information

Supplementary Notes

Pooled and Panel Data Models: Pooling observations over all crops, farms, and years suggests that being organic leads to a ~96% decrease in total pesticide active ingredients (kg ha-1 AI), after accounting for soil quality, and farm and field size covariates (Supplementary Table 2). We inverse hyperbolic sine transform pesticide use (and field, farm size) to accommodate zero values and improve normality of the residuals. For level terms (organic, soil quality), coefficients can be converted to semi-elasticities by exponentiating $100(e^{\beta} - 1)$, while inverse hyperbolic sine (ihs) transformed coefficients (field, farm size) can be interpreted as elasticities (% change − % change) directly. Subsetting the sample to just observations from farms that grow both organic and conventional crops within the study period and crops that are grown both organically and conventionally reduces the sample size by around ⅔, but has minimal effect on the organic coefficient (Supplementary Table 2).

Including farm-by-crop family or farm, crop family, and year effects using a within-estimator panel approach (i.e., "fixed effects" in causal inference terminology, which differs from biostatistical use of the term) leads to a slight reduction in the organic coefficient, but there remains a ~91% decrease relative to conventional (Supplementary Table 2). We find very similar point estimates for the random effects model with random intercepts for farm-by-crop family (Supplementary Table 2) or for crossed farm and crop family random effects (Supplementary Table 3). Coefficients on soil quality and field size are substantially smaller in magnitude in either panel data model (within-estimator or random effects) relative to the pooled ordinary least squares (OLS). Across specifications, larger field and farm size and better soil quality lead to an increase in pesticide use. Due to collinearity with the farm-by-crop family effects in the within estimator, farm size is dropped from that panel model. Similarly, observations with ambiguous crop family (e.g., uncultivated agriculture, ornamental plants) were dropped in both panel model analyses and in the pooled OLS with clustered standard errors (clustered at the farm-by-crop family) due to missing data.

Overall, the within-estimator and random effects models produce comparable results for organic and other coefficients. We proceed with farm-by-crop family effects rather than crossed random effects based on AIC/BIC and computational feasibility with cluster robust standard errors (see methods). Choice of random effects had little impact on our organic covariate, though including farmer effects did, unsurprisingly, affect the coefficient for farm size in particular (Supplementary Table 3).

Supplementary Figures

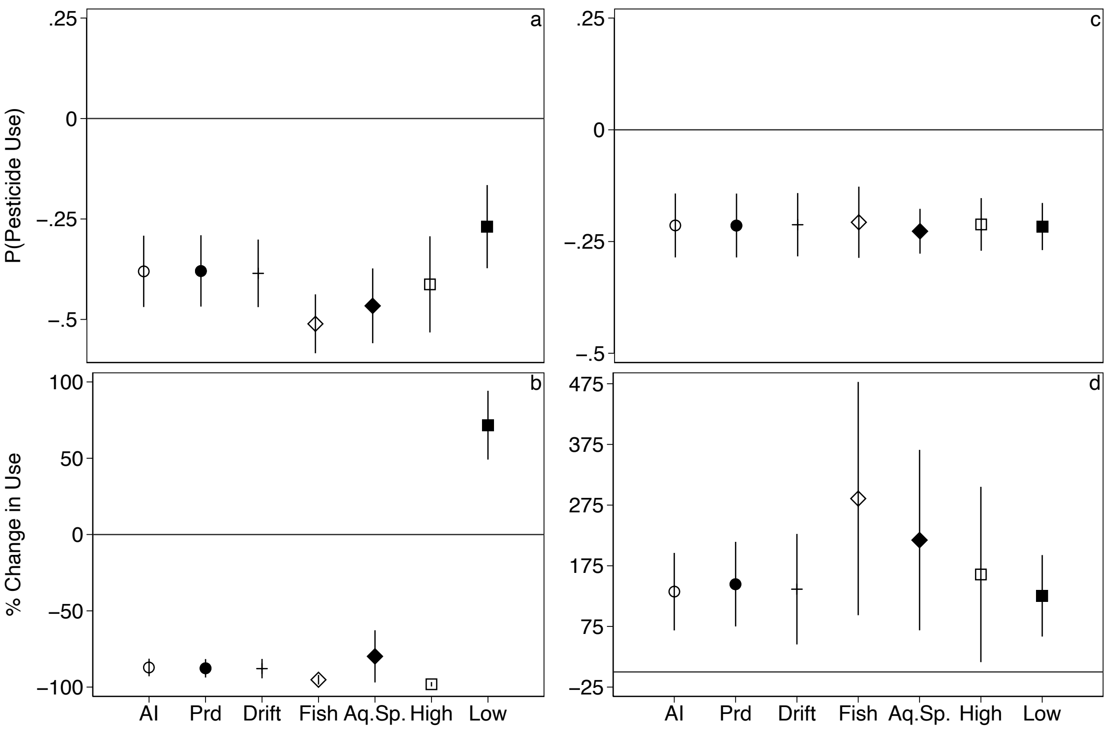

Supplementary Figure 1. Double hurdle models for different pesticide use outcomes for Carrots (**a,b**) and Grapes (**c,d**). The x-axis indicates different measures of pesticide use outcomes that were reasonably common for these crops (see methods): kg ha^{-1} active ingredients (AI), kg ha^{-1} products (Prd), kg $ha^{-1}$of products with propensity to drift (Drift), kg ha^{-1} products of potential hazard to aquatic species (Aq. Sp.), as well as products of higher (EPA signal word 1–2) and lower (EPA signal word 3–4) acute toxicity (High, Low). Across all outcomes and for both crops, organic fields have a significantly lower probability of using pesticides (**a,c**). However, for fields that are sprayed, there are very different effects of organics on carrots (**b**) than grapes (**d**). All models include covariates for field size, farm size, and soil quality. For all crop-specific models, we include year random intercepts and heteroskedasticity robust standard errors. Symbols at the center of the error bars indicate point estimates (mean) and error bars represent the 95 % CI. Number of observations for panel **a**: 4,289; number of observations for panel **b**: 2,766 (AI), 2,769 (Prd), 2,754 (Drift), 2,386 (Fish), 2,205 (Aq. Sp.), 2,082 (High), 2,284 (Low). Number of observations for panel **c**: 8,760; number of observations for panel **d**: 7,678 (AI), 7,680 (Prd), 7,666 (Drift), 7,311 (Fish), 7,436 (Aq. Sp.), 7,334 (High), 7,548 (Low). Coefficient estimates for all covariates are provided in Supplementary Tables 7-8.

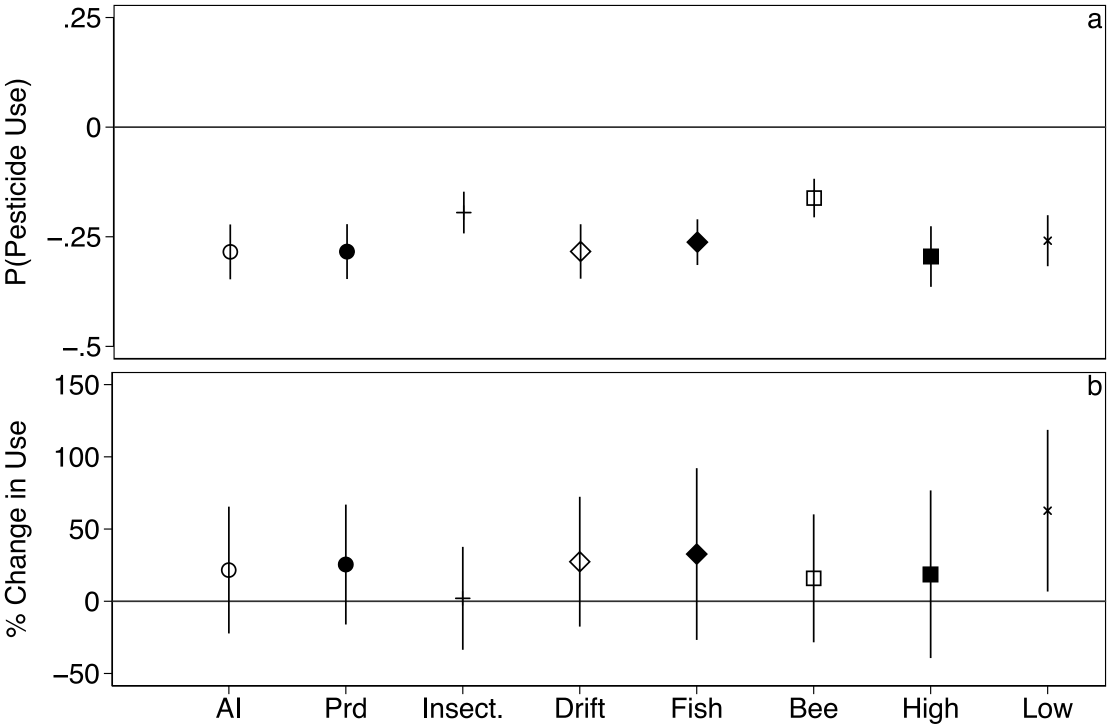

Supplementary Figure 2. Lognormal hurdle models for 2017-2019 estimating the change in the probability of pesticide use (**a**) and the percent change in pesticide use for fields with positive use (**b**) for organic relative to conventional fields, including as "organic" observations self-reporting organic commodities in addition to PUR Organic observations. Including observations with self-reported organic status (available in 2017-2019 only) slightly shifts our coefficients relative to models that omit these observations (Supplementary Figure 3). The x-axis indicates different measures of pesticide use outcomes: kg ha^{-1} active ingredients (AI), kg ha^{-1} products (Prd), kg ha^{-1} of products targeting insect pests only (Insect), kg ha^{-1} of products with propensity to drift (Drift), kg ha^{-1} products of potential hazard to fish and bees (Fish, Bee), as well as products of higher (EPA signal word 1–2) and lower (EPA signal word 3–4) acute toxicity (High, Low). All models include covariates for field size, farm size, and soil quality as well farm-by-crop family random effects. Symbols at the center of the error bars indicate point estimates (mean) and error bars represent the 95 % CI. All models include cluster robust standard errors clustered at the farm-crop family level. Number of observations in panel A: 40,002; number of observations in panel B: 30,212 (AI), 30,247 (Prd), 23,253 (Insect.) 29,974 (Drift), 26,898 (Fish), 21,299 (Bee), 28,124 (High), 28,848 (Low). Coefficient estimates for all covariates are provided in Supplementary Table 12.

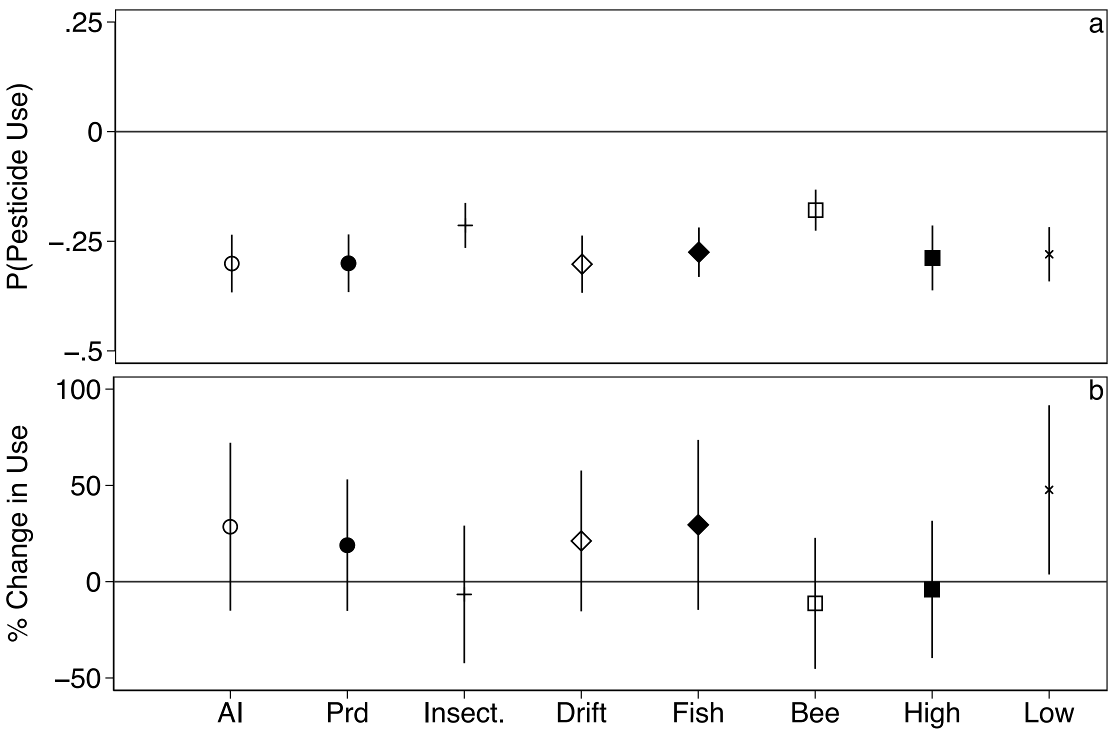

Supplementary Figure 3. Lognormal hurdle models for 2017-2019 estimating the change in the probability of pesticide use (**a**) and the percent change in pesticide use for fields with positive use (**b**) for organic relative to conventional fields including as "organic" only PUR Organic observations. Ignoring observations with self-reported organic status results in slightly shifted coefficient estimates (Supplementary Figure 2). The x-axis indicates different measures of pesticide use outcomes: kg ha^{-1} active ingredients (AI), kg ha^{-1} products (Prd), kg ha^{-1} of products targeting insect pests only (Insect), kg ha^{-1} of products with propensity to drift (Drift), kg ha^{-1} products of potential hazard to fish and bees (Fish, Bee), as well as products of higher (EPA signal word 1–2) and lower (EPA signal word 3–4) acute toxicity (High, Low). All models include covariates for field size, farm size, and soil quality as well farm-by-crop family random effects. Symbols at the center of the error bars indicate point estimates (mean) and error bars represent the 95 % CI. All models include cluster robust standard errors clustered at the farm-crop family level. Number of observations in panel A: 40,002; number of observations in panel B: 30,212 (AI), 30,247 (Prd), 23,253 (Insect.) 29,974 (Drift), 26,898 (Fish), 21,299 (Bee), 28,124 (High), 28,848 (Low). Coefficient estimates for all covariates are provided in Supplementary Table 13.

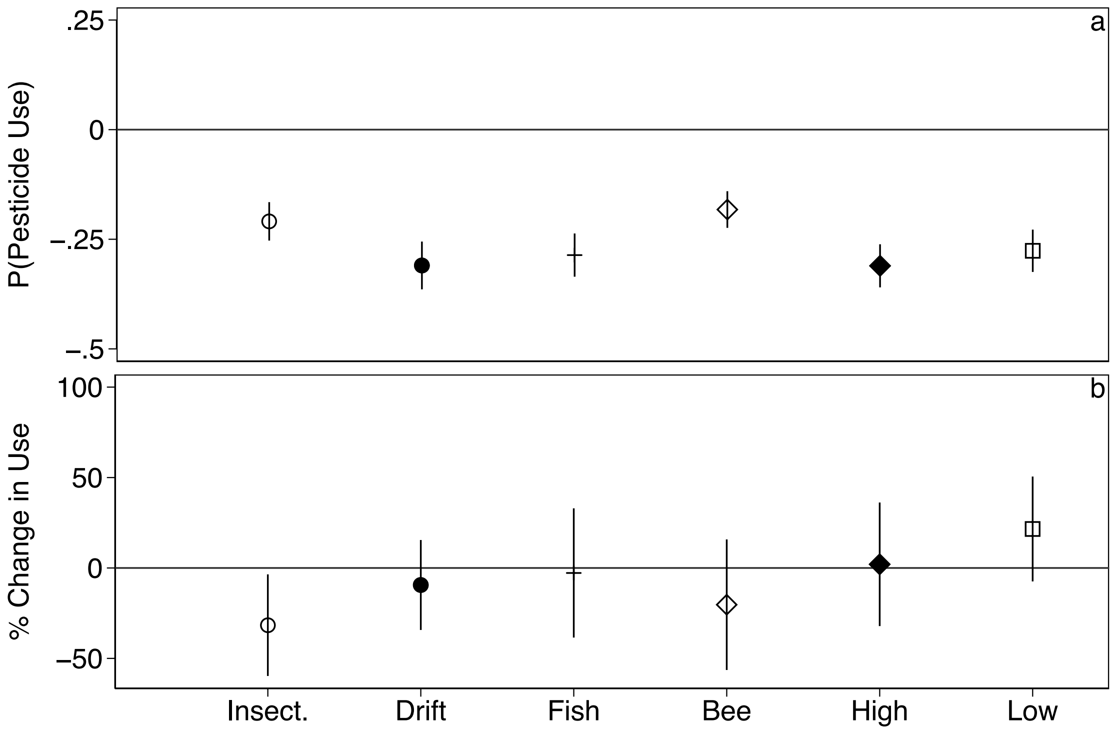

Supplementary Figure 4. Lognormal hurdle models estimating the change in the probability of pesticide use (**a**) and the percent change in pesticide use for fields with positive use (**b**) for organic relative to conventional fields, using kg of active ingredients rather than kg of products for different pesticide use outcomes. Coefficient estimates for models using kg of active ingredients (AI) are very similar to the main text models using kg of products for the different pesticide outcomes. The x-axis indicates different measures of pesticide use outcomes: kg ha^{-1} of active ingredients targeting insect pests only (Insect), kg ha^{-1} of AI with propensity to drift (Drift), kg ha^{-1} AI of potential hazard to fish and bees (Fish, Bee), as well as AI of higher (EPA signal word 1–2) and lower (EPA signal word 3–4) acute toxicity (High, Low). All models include covariates for field size, farm size, and soil quality as well farm-by-crop family random effects. Symbols at the center of the error bars indicate point estimates (mean) and error bars represent the 95 % CI. All models include cluster robust standard errors clustered at the farm-crop family level. Number of observations in panel A: 91,926; number of observations in panel B: 52,606 (Insect.), 67,967 (Drift), 60,638 (Fish), 48,254 (Bee), 55,106 (High), 64,114 (Low). Coefficient estimates for all covariates are provided in Supplementary Table 14.

Supplementary Tables

Supplementary Table 1. Analysis predicting soil quality as a function of organic and conventional practice.

	(1) All	(2) Subsample	(3) All, Factor X-Chem	(4) All, Factor X-Hydro
Organic	-0.191** (0.055)	-0.133* (0.056)	0.002~ (0.001)	0.013 (0.008)
Crop dummy	X	X	X	X
Subsample, overlapping commodity & permit		X		
Observations	99,214	38,835	97,025	11,962

Note: Organic fields have higher soil quality (where 1 is good, 6 is poor) even after controlling for crop type (1) and when comparing the subset of fields grown by farmers and of crop types that have both organic and conventional production (2). The dynamic component (factor X) of the Storie Index could conceivably be influenced by on-farm practices, introducing potential endogeneity concerns. However, the underlying factors (X-Chem, X-Hydro) are not very different between organic and conventional (3–4), after controlling for crop type, suggesting these dynamic factors are not driving the difference in soil quality we observe for organic vs conventional. Models include dummy variables for crop type and cluster robust standard errors clustered at crop type. Differences in number of observations (1,3,4) is due to missing values in the outcome variables. ~, *, ** indicating $p<0.1$, $p<0.05$, $p<0.01$, respectively, based on two-tailed t-test with $\alpha = 0.05$ and no adjustments for multiple comparisons. Exact p-values, rounded to the third decimal place, are: $p = 0.001$ (1), $p = 0.019$ (2), $p = 0.098$ (3), $p = 0.121$ (4).

Supplementary Table 2. Different linear model specifications.

VARIABLES	(1) OLS	(2) Subsample	(3) FE	(4) FE	(5) RE
Organic	-3.179**	-3.537**	-2.570**	-2.384**	-2.411**
	(0.285)	(0.294)	(0.256)	(0.268)	(0.257)
IHS(Field Size)	0.384**	0.269**	0.171**	0.140**	0.179**
	(0.076)	(0.103)	(0.033)	(0.032)	(0.031)
Soil Quality	-0.575**	-0.303**	-0.063*	-0.065**	-0.072**
	(0.070)	(0.113)	(0.025)	(0.023)	(0.022)
IHS(Farm Size)	0.245**	0.401**			0.130*
	(0.062)	(0.090)			(0.057)
Farm			X		
Crop family			X		
Year			X		
Farm x crop family				X	X
Subsample, overlapping commodity & permit		X			
Observations	91,926	35,075	91,823	91,590	91,926

Note: Pooled OLS (OLS), observations with overlapping farm and crop for organic and conventional (subsample), within (or fixed effect, FE) and random effect (RE) models predicting pesticide use as a function of organic status and field size, farm size and soil quality. Pesticide use (products kg ha^{-1}), field size and farm size are IHS transformed. Coefficients on ihs transformed variables can be interpreted as elasticities (%-% change). Coefficients on non-transformed variables (organics, soil quality) can be interpreted as $100(e^{\beta} - 1)$ percent change in pesticide use. FE and RE models include effects indicated by X. Across all models, being organic results in a 91–97% decrease in pesticide use. Models include cluster robust errors clustered at farm-by-crop family with ~, *, ** indicating $p<0.1$, $p<0.05$, $p<0.01$, respectively, based on two-tailed t-test with $\alpha = 0.05$ and no adjustments for multiple comparisons. Observations vary due to missing data on crop family, or due to a lack of within variation in the specified models (3,4).

Supplementary Table 3. Model comparison of different random effects (random intercepts) specifications.

	(1)	(2)	(3)	(4)	(5)
Organic	-2.711**	-2.308**	-2.601**	-2.411**	-2.411**
	(0.043)	(0.043)	(0.061)	(0.060)	(0.257)
IHS(Field Size)	0.302**	0.208**	0.187**	0.179**	0.179**
	(0.012)	(0.011)	(0.013)	(0.013)	(0.031)
Soil Quality	-0.031**	-0.041**	-0.063**	-0.072**	-0.072**
	(0.010)	(0.009)	(0.012)	(0.012)	(0.022)
IHS(Farm Size)	0.352**	0.319**	0.206**	0.130**	0.130*
	(0.007)	(0.007)	(0.026)	(0.024)	(0.057)
Farmer			X		
Crop		X			
Crop family	X		X		
Year	X	X			
Farmer x crop family				X	X
Clustered SEs					X
AIC	459,068	487,244	444,127	438,225	438,225
BIC	459,143	487,320	444,202	438,291	438,291
Observations	91,926	99,213	91,926	91,926	91,926

Note: Different random effects models, with random coefficients as indicated by the X. Models with more than one effect indicate models with crossed random effects. The specification including farm-by-crop family effects (model 4) had the lowest AIC, BIC. Model 5 is the same as model 4, but accounts for heteroskedasticity and potential intragroup correlation of the errors using cluster robust standard errors clustered at the farm-by-crop family level. Throughout, soil quality is based on the Storie Index where 1 is good soil and 6 is poor. As elsewhere, ~, *, ** indicating $p<0.1$, $p<0.05$, $p<0.01$, based on two-tailed t-test with $\alpha = 0.05$ and no adjustments for multiple comparisons. Observations vary due to missing data on crop family.

Supplementary Table 4. Summary Statistics.

		Total fields	Average field size (ha)	Average soil quality (1 = high, 6 = low)	Average active ingredients applied (kg ha^{-1})	Average pesticide product applied (kg ha^{-1})
All Fields	Conv	90,439	32.03 ± 0.11	1.87 ± <0.01	26.91 ± 0.27	47.62 ± 0.43
	Org	9,094	17.91 ± 0.17	1.37 ± 0.01	8.04 ± 0.27	17.10 ± 0.48
All fields that spray (use > 0 kg pesticide products)	Conv	66,304	33.16 ± 0.13	1.81 ± <0.01	36.71 ± 0.37	64.96 ± 0.57
	Org	3,993	16.99 ± 0.23	1.34 ± 0.01	18.30 ± 0.58	38.94 ± 0.98
Carrots	Conv	2,886	26.88 ± 0.25	1.35 ± 0.01	127.90 ± 3.61	253.46 ± 7.28
	Org	1,403	29.22 ± 0.36	1.29 ± 0.01	11.67 ± 0.60	19.90 ± 0.98
Carrots (spray)	Conv	2,148	27.18 ± 0.28	1.35 ± 0.01	171.85 ± 4.47	340.54 ± 9.04
	Org	621	28.77 ± 0.49	1.27 ± 0.02	26.37 ± 1.10	44.96 ± 1.74
Grapes	Conv	8,443	27.88 ± 0.34	1.54 ± 0.01	59.83 ± 1.96	98.20 ± 2.38
	Org	317	27.73 ± 1.37	1.52 ± 0.05	65.38 ± 4.16	124.91 ± 7.55
Grapes (spray)	Conv	7,473	28.82 ± 0.37	1.55 ± 0.01	67.60 ± 2.20	110.95 ± 2.65
	Org	207	31.57 ± 1.82	1.61 ± 0.07	100.12 ± 4.88	191.29 ± 8.50
Onions	Conv	985	22.05 ± 0.61	1.59 ± 0.03	21.04 ± 2.69	39.90 ± 4.98
	Org	141	23.63 ± 1.20	1.21 ± 0.03	26.65 ± 4.39	38.46 ± 5.28
Onions (spray)	Conv	740	23.46 ± 0.75	1.59 ± 0.04	28.01 ± 3.55	53.21 ± 6.56
	Org	74	23.35 ± 1.75	1.23 ± 0.05	50.78 ± 7.33	73.29 ± 8.19
Oranges	Conv	4,520	24.05 ± 0.46	1.43 ± 0.01	50.06 ± 0.89	92.05 ± 1.33
	Org	141	26.04 ± 1.84	1.33 ± 0.05	33.16 ± 4.54	44.82 ± 5.13
Oranges (spray)	Conv	4,230	24.39 ± 0.48	1.43 ± 0.01	53.49 ± 0.93	98.37 ± 1.37
	Org	93	28.31 ± 2.33	1.27 ± 0.04	50.27 ± 6.19	67.95 ± 6.61
Potatoes	Conv	2,315	29.07 ± 0.37	1.45 ± 0.01	46.80 ± 1.96	94.13 ± 4.25
	Org	489	28.09 ± 0.65	1.30 ± 0.02	1.61 ± 0.17	4.61 ± 0.49
Potatoes (spray)	Conv	1,886	29.36 ± 0.40	1.45 ± 0.01	57.45 ± 2.34	115.54 ± 5.09
	Org	173	27.33 ± 0.81	1.30 ± 0.03	4.54 ± 0.41	13.02 ± 1.13

Note: Mean ± standard error (sd/$\sqrt{n}$) for field size, soil quality, and pesticide use for all fields and five commonly grown crops, separated by organic ("Org") and conventional ("Conv"). Summary values are shown for all fields within each of the aforementioned groups, and for the

subset of fields within those groups that use any type of pesticide (“spray”), reflecting summary statistics for fields used in the second hurdle model.

Supplementary Table 5. Lognormal hurdle models for different pesticide use metrics.

VARIABLES	(1)<br>All AI	(2)<br>All Prd	(3)<br>Drift	(4)<br>Insect. Only	(5)<br>Fish	(6)<br>Bees	(7)<br>EPA High	(8)<br>EPA Low
(A) Hurdle 1, binary								
Organic	-0.311**	-0.311**	-0.311**	-0.209**	-0.286**	-0.182**	-0.298**	-0.284**
	(0.028)	(0.028)	(0.028)	(0.022)	(0.025)	(0.021)	(0.030)	(0.025)
ln(Field Size)	0.029**	0.029**	0.028**	0.024**	0.030**	0.021**	0.032**	0.032**
	(0.003)	(0.003)	(0.004)	(0.004)	(0.003)	(0.003)	(0.003)	(0.004)
Soil Quality	-0.008**	-0.008**	-0.008**	-0.010**	-0.011**	-0.008**	-0.005~	-0.009**
	(0.002)	(0.002)	(0.002)	(0.003)	(0.003)	(0.003)	(0.002)	(0.003)
ln(Farm Size)	0.021**	0.021**	0.021**	-0.003	0.016**	-0.000	0.020**	0.017**
	(0.005)	(0.005)	(0.005)	-0.209**	(0.005)	(0.008)	(0.005)	(0.006)
Avg. Partial Effects	X	X	X	X	X	X	X	X
Farmer x family, Yr RE	X	X	X	X	X	X	X	X
Observations	91,926	91,926	91,926	91,926	91,926	91,926	91,926	91,926
(B) Hurdle 2, For Kg >0								
Organic	-0.113	-0.063	-0.007	-0.181	0.048	-0.122	-0.314*	0.249*
	(0.133)	(0.125)	(0.122)	(0.175)	(0.153)	(0.174)	(0.153)	(0.108)
ln(Field Size)	0.004	-0.025	-0.021	-0.002	-0.030~	-0.023	-0.032~	-0.026
	(0.019)	(0.016)	(0.018)	(0.020)	(0.017)	(0.018)	(0.019)	(0.017)
Soil Quality	-0.022~	-0.015	-0.012	-0.010	-0.028*	-0.021	-0.011	-0.015
	(0.012)	(0.009)	(0.010)	(0.013)	(0.013)	(0.016)	(0.011)	(0.011)
ln(Farm Size)	-0.092~	-0.107*	-0.124**	-0.147**	-0.069	-0.091**	-0.082~	-0.123**
	(0.050)	(0.042)	(0.047)	(0.040)	(0.047)	(0.029)	(0.046)	(0.046)
Farmer x family	X	X	X	X	X	X	X	X
Observations	68,704	68,816	67,988	52,606	60,653	48,254	61,883	65,593

Notes: Lognormal double hurdle models estimating the effect of organic on the decision to spray (A) and the decision of how much to spray on fields with positive pesticide use (B) across different pesticide use metrics: all active ingredients (All AI), all products (All Prd), chemicals prone to drift (Drift), chemicals functioning as insecticides only (Insect. Only), chemicals of potential hazard to fish

and bees based on the CDPR Product Database, as well as chemicals with high and low acute toxicity based on the EPA signal word. We include farm-by-crop family random effects. For all double hurdle models, we use the natural log of pesticide use metrics as the outcome and the natural log of field and farm size (in contrast to ihs transformation in Supplementary Table 2–3, see text). Throughout, in the first hurdle, which is estimated using a random effect probit, we report the average partial effects. Models include cluster robust errors clustered at farm-by-crop family with ~, *, ** indicating $p<0.1$, $p<0.05$, $p<0.01$, respectively, based on two-tailed t-test with $\alpha = 0.05$ and no adjustments for multiple comparisons.

Supplementary Table 6. Double hurdle models for relatively widely grown organic and conventional crops.

VARIABLES	(1) Carrot	(2) Grape	(3) Orange	(4) Potato	(5) Dry Onion	(6) Average
(A) Hurdle 1, binary						
Organic	-0.380**	-0.214**	-0.265**	-0.508**	-0.268**	-0.433**
	(0.045)	(0.036)	(0.053)	(0.030)	(0.051)	(0.013)
ln(Field Size)	0.027	0.022**	0.021**	0.031**	0.061**	0.021**
	(0.019)	(0.005)	(0.005)	(0.012)	(0.014)	(0.003)
Soil Quality	-0.020	-0.003	-0.002	0.003	-0.009	0.004
	(0.016)	(0.004)	(0.010)	(0.023)	(0.021)	(0.004)
ln(Farm Size)	0.034*	0.031**	0.017**	0.036**	0.022*	0.012**
	(0.014)	(0.004)	(0.002)	(0.009)	(0.009)	(0.003)
Avg. Partial Effects	X	X	X	X	X	X
Yr RE	X	X	X	X	X	X
Observations	4,289	8,760	4,654	2,804	1,126	21, 633
(B) Hurdle 2, For Kg >0						
Organic	-2.050**	0.843**	-1.022**	-1.658**	0.429	-0.835**
	(0.228)	(0.140)	(0.295)	(0.265)	(0.355)	(0.178)
ln(Field Size)	0.052	0.016	0.104*	-0.155~	-0.072	0.041
	(0.248)	(0.069)	(0.046)	(0.089)	(0.104)	(0.059)
Soil Quality	0.046	0.097**	0.002	0.255**	-0.155~	0.072**
	(0.054)	(0.035)	(0.049)	(0.092)	(0.082)	(0.022)
ln(Farm Size)	0.301*	0.121**	0.137**	0.060	-0.007	0.067**
	(0.123)	(0.037)	(0.036)	(0.091)	(0.041)	(0.011)
Yr RE	X	X	X	X	X	X
Observations	2,766	7,678	4,316	2,059	814	17,633

Note: Lognormal double hurdle models predicting kg ha^{-1} of active ingredients for different widely grown crops and the average (i.e. pooled model) across this subset of crops. These crops had at least 100 organic fields and at least 15,000 ha of conventional production, and had some observations with positive pesticides on organic fields. We include year random effects. For all double hurdle models, we use the natural log of pesticide use metrics as the outcome and the natural log of field and farm size. In the first hurdle, which is estimated using a random effects probit model, we report the average partial effects. Corresponds to Figure 3. For crop-specific models, we include year random effects (intercepts) and heteroskedasticity robust standard errors. As elsewhere, ~, *, ** indicating p<0.1, p<0.05, p<0.01, based on two-tailed t-test with α = 0.05 and no adjustments for multiple comparisons.

Supplementary Table 7. Double hurdle models for different pesticide use metrics for carrots.

VARIABLES	(1)<br>All AI	(2)<br>All Prd	(3)<br>Drift	(4)<br>Fish	(5)<br>Aq. Sp	(6)<br>EPA High	(7)<br>EPA Low
(A) Hurdle 1, binary							
Organic	-0.380**	-0.379**	-0.385**	-0.511**	-0.466**	-0.413**	-0.269**
	(0.045)	(0.045)	(0.043)	(0.037)	(0.047)	(0.061)	(0.053)
ln(Field Size)	0.027	0.028	0.029	0.024	0.017	0.043	0.021
	(0.019)	(0.019)	(0.018)	(0.020)	(0.019)	(0.030)	(0.019)
Soil Quality	-0.020	-0.021	-0.018	-0.020	-0.020	-0.001	-0.021
	(0.016)	(0.016)	(0.016)	(0.014)	(0.018)	(0.013)	(0.016)
ln(Farm Size)	0.034*	0.034*	0.033*	0.022	0.022	0.034*	0.045**
	(0.014)	(0.014)	(0.014)	(0.015)	(0.014)	(0.016)	(0.013)
Avg. Partial Effects	X	X	X	X	X	X	X
Year RE	X	X	X	X	X	X	X
Observations	4,289	4,289	4,289	4,289	4,289	4,289	4,289
(B) Hurdle 2, For Kg >0							
Organic	-2.050**	-2.092**	-2.110**	-3.030**	-1.601**	-3.977**	0.540**
	(0.228)	(0.249)	(0.268)	(0.335)	(0.433)	(0.394)	(0.067)
ln(Field Size)	0.052	0.043	0.050	0.089	-0.097	-0.022	-0.282~
	(0.248)	(0.239)	(0.244)	(0.308)	(0.164)	(0.285)	(0.148)
Soil Quality	0.046	-0.008	-0.016	-0.023	-0.047	-0.151	0.119*
	(0.054)	(0.053)	(0.054)	(0.062)	(0.072)	(0.101)	(0.057)
ln(Farm Size)	0.301*	0.316**	0.328**	0.370**	0.154	0.298*	0.172**
	(0.123)	(0.115)	(0.119)	(0.111)	(0.110)	(0.124)	(0.063)
Year RE	X	X	X	X	X	X	X
Observations	2,766	2,769	2,754	2,386	2,205	2,082	2,284

Notes: Lognormal double hurdle models estimating the effect of organic on the decision to spray (A) and the decision of how much to spray on fields with positive pesticide use (B) across different pesticide use metrics for carrots: all active ingredients (All AI), all products (All Prd), chemicals prone to drift (Drift), chemicals of potential hazard to fish and aquatic species based on the CDPR

Product Database, as well as chemicals with high and low acute toxicity based on the EPA signal word. For all crop-specific models, we include year random intercepts and heteroskedasticity robust standard errors. As elsewhere, ~, *, ** indicating $p<0.1$, $p<0.05$, $p<0.01$, based on two-tailed t-test with $\alpha = 0.05$ and no adjustments for multiple comparisons.

Supplementary Table 8. Double hurdle models for different pesticide use metrics for grapes.

VARIABLES	(1)<br>All AI	(2)<br>All Prd	(3)<br>Drift	(4)<br>Fish	(5)<br>Aq. Sp	(6)<br>EPA High	(7)<br>EPA Low
(A) Hurdle 1, binary							
Organic	-0.214**	-0.214**	-0.212**	-0.207**	-0.227**	-0.212**	-0.216**
	(0.036)	(0.036)	(0.036)	(0.041)	(0.026)	(0.030)	(0.027)
ln(Field Size)	0.022**	0.022**	0.022**	0.030**	0.026**	0.031**	0.023**
	(0.005)	(0.005)	(0.005)	(0.006)	(0.007)	(0.002)	(0.006)
Soil Quality	-0.003	-0.003	-0.004	-0.005	-0.006	-0.006	-0.003
	(0.004)	(0.004)	(0.004)	(0.004)	(0.004)	(0.004)	(0.005)
ln(Farm Size)	0.031**	0.031**	0.031**	0.030**	0.031**	0.036**	0.030**
	(0.004)	(0.004)	(0.004)	(0.005)	(0.005)	(0.005)	(0.005)
Avg. Partial Effects	X	X	X	X	X	X	X
Year RE	X	X	X	X	X	X	X
Observations	8,760	8,760	8,760	8,760	8,760	8,760	8,760
(B) Hurdle 2, For Kg >0							
Organic	0.843**	0.895**	0.860**	1.350**	1.155**	0.958**	0.813**
	(0.140)	(0.145)	(0.197)	(0.254)	(0.239)	(0.283)	(0.152)
ln(Field Size)	0.016	-0.022	-0.022	-0.024	-0.007	-0.002	-0.048
	(0.069)	(0.063)	(0.065)	(0.037)	(0.054)	(0.080)	(0.061)
Soil Quality	0.097**	0.068*	0.077*	0.110**	0.094**	0.092*	0.087*
	(0.035)	(0.032)	(0.033)	(0.036)	(0.036)	(0.037)	(0.035)
ln(Farm Size)	0.121**	0.137**	0.133**	0.144**	0.105**	0.186**	0.088**
	(0.037)	(0.029)	(0.027)	(0.034)	(0.031)	(0.030)	(0.031)
Year RE	X	X	X	X	X	X	X
Observations	7,678	7,680	7,666	7,311	7,436	7,334	7,548

Notes: Lognormal double hurdle models estimating the effect of organic on the decision to spray (A) and the decision of how much to spray on fields with positive pesticide use (B) across different pesticide use metrics for grapes: all active ingredients (All AI), all

products (All Prd), chemicals prone to drift (Drift), chemicals of potential hazard to fish and aquatic species based on the CDPR Product Database, as well as chemicals with high and low acute toxicity based on the EPA signal word. For all crop-specific models, we include year random intercepts and heteroskedasticity robust standard errors. As elsewhere, ~, *, ** indicating $p<0.1$, $p<0.05$, $p<0.01$, based on two-tailed t-test with $\alpha = 0.05$ and no adjustments for multiple comparisons.

Supplementary Table 9. Double hurdle models accounting for yield gap.

VARIABLES	(1)<br>All AI	(2)<br>All Prd	(3)<br>Drift	(4)<br>Insect. Only	(5)<br>Fish	(6)<br>Bees	(7)<br>EPA High	(8)<br>EPA Low
(A) Hurdle 1, binary								
Organic	-0.311**	-0.311**	-0.311**	-0.209**	-0.286**	-0.182**	-0.298**	-0.284**
	(0.028)	(0.028)	(0.028)	(0.022)	(0.025)	(0.021)	(0.030)	(0.025)
ln(Field Size)	0.029**	0.029**	0.028**	0.024**	0.030**	0.021**	0.032**	0.032**
	(0.003)	(0.003)	(0.004)	(0.004)	(0.003)	(0.003)	(0.003)	(0.004)
Soil Quality	-0.008**	-0.008**	-0.008**	-0.010**	-0.011**	-0.008**	-0.005~	-0.009**
	(0.002)	(0.002)	(0.002)	(0.003)	(0.003)	(0.003)	(0.002)	(0.003)
ln(Farm Size)	0.021**	0.021**	0.021**	-0.003	0.016**	-0.000	0.020**	0.017**
	(0.005)	(0.005)	(0.005)	(0.008)	(0.005)	(0.008)	(0.005)	(0.006)
Avg. Partial Effects	X	X	X	X	X	X	X	X
Farmer x family, Yr RE	X	X	X	X	X	X	X	X
Observations	91,926	91,926	91,926	91,926	91,926	91,926	91,926	91,926
(B) Hurdle 2, For Kg >0								
Organic	0.012	0.063	0.116	-0.065	0.168	-0.006	-0.198	0.373**
	(0.131)	(0.124)	(0.121)	(0.177)	(0.152)	(0.175)	(0.151)	(0.107)
ln(Field Size)	0.004	-0.025	-0.021	-0.002	-0.030~	-0.023	-0.032~	-0.026
	(0.019)	(0.016)	(0.018)	(0.020)	(0.017)	(0.018)	(0.019)	(0.017)
Soil Quality	-0.022~	-0.015	-0.012	-0.010	-0.028*	-0.021	-0.011	-0.015
	(0.012)	(0.009)	(0.010)	(0.013)	(0.013)	(0.016)	(0.011)	(0.011)
ln(Farm Size)	-0.092~	-0.107*	-0.124**	-0.147**	-0.069	-0.091**	-0.082~	-0.122**
	(0.050)	(0.042)	(0.047)	(0.040)	(0.047)	(0.029)	(0.046)	(0.046)
Farmer x family, Yr RE	X	X	X	X	X	X	X	X
Observations	68,704	68,816	67,988	52,606	60,653	48,254	61,883	65,593

Notes: Double hurdle models adjusting pesticide use (kg ha^{-1}) for yield gaps based on Ponisio et al.[1] (see text). The decision to spray (A) and the decision of how much to spray on fields with positive pesticide use (B) across different pesticide use metrics: all active ingredients (All AI), all products (All Prd), chemicals prone to drift (Drift), chemicals functioning as insecticides only (Insect. Only),

chemicals of potential hazard to fish and bees based on the CDPR Product Database, as well as chemicals with high and low acute toxicity based on the EPA signal word. We include farm-by-crop family random effects and cluster robust standard errors clustered at the farm-by-crop family level. As elsewhere, ~, *, ** indicating $p<0.1$, $p<0.05$, $p<0.01$, based on two-tailed t-test with $\alpha = 0.05$ and no adjustments for multiple comparisons.

Supplementary Table 10. Ecotoxicological data for chemicals representing about 50% of use (by product weight) for organic and conventional fields.

Field status	Product name	EPA registration #	Product status	Fraction of total products used	Active Ingredient (AI)	% AI by wt	CAS #	Mammal Acute Oral LD_{50} (mg kg^{-1})	Birds - Acute LD_{50} (mg kg^{-1})	Fish - Acute 96h LC_{50} (mg l^{-1})	Aquatic inverts - Acute 48h EC_{50} (mg l^{-1})	Freshwater Algae - Acute 72 hr EC_{50} (mg l^{-1})
Org	Thiolux	34704-1079	Org	0.089	Sulfur	80	7704-34-9	> 2000	> 2000	> 0.063	> 0.063	> 0.063
Org	Lime Sulfur Solution	61842-30	Org	0.083	Calcium Polysulfide	29	1344-81-6	1343	560	> 2.86	> 6.6	>12.6
Org	Serenade ASO	264-1152	Org	0.071	Bacillus subtilis strain QST 713	1.34	NA	Low toxicity	> 5000	No significa nt risk	No significant risk	No record found
Org	Surround WP Crop Protectant	61842-18	Org	0.070	Kaolin	95	1332-58-7	> 5000	> 5000	> 2500	> 2500	>100
Org	Sonata	69592-13	Org	0.039	Bacillus pumilus strain qst2808	1.38	NA	> 4.1 x 109	> 7.0 x 10^{10}	2.6 x 107	6.2 x 108	No record found
Org	Suppress EC	51517-9	Org	0.036	Caprylic acid, capric acid	47, 32	0124-07-02, 334-48-5	> 2000	>2250	35	16.9	43.73
Org	Sonata	264-1153	Org	0.036	Bacillus pumilus strain qst2808	1.38	NA	> 4.1 x 10^{9}	> 7.0 x 10^{10}	2.6 x 10^{7}	6.2 x 10^{8}	No record found
Org	Aza-Direct	71908-1-10163	Org	0.036	Azadirachtin	1.2	11141-17-6	> 5000	> 2250	37	9.3	> 5.76
Org	Cosavet DF	70905-1	Org	0.034	Sulphur	80	7704-34-9	> 2000	> 2000	> 0.063	> 0.063	> 0.063
Conv	Sectagon-K54	61842-7	Conv	0.060	Potassium methyldithiocarbamate	54	137-41-7	630	> 211	> 54.0	> 4.6	0.56
Conv	K-Pam HL	5481-483	Conv	0.055	Potassium methyldithiocarbamate	54	137-41-7	630	> 211	> 54.0	> 4.6	0.56
Conv	Vapam HL	5481-468	Conv	0.049	Sodium methyldithiocarbamate	42	137-42-8	896	211	> 0.175	0.99	No record found
Conv	Telone II	62719-32	Conv	0.042	1,3-	97.5	542-75-6	150	92	2.78	3.58	6.4

					Dichloropropene							
Conv	Round-up Powermax Herbicide	524-549	Conv	0.039	Potassium salt of glyphosate	51.2	70919-12-1	> 2000	> 2241	> 1227	> 1227	> 35.0
Conv	Sectagon 42	61842-6	Conv	0.035	Potassium methyldithiocarbamate	54	137-41-7	630	> 211	> 54.0	> 4.6	0.56
Conv	Microthiol Disperss	70506-187	Org	0.025	Sulfur	80	7704-34-9	> 2000	> 2000	> 0.063	> 0.063	> 0.063
Conv	Special Electric Refined Super-Adhesive Dusting Sulfur	2935-503	Org	0.018	Sulfur	90-100	7704-34-9	> 2000	> 2000	> 0.063	> 0.063	> 0.063
Conv	IAP Summer 415 Spray oil	71058-5	Org	0.017	Mineral oil	98.5	8042-47-5	3340	> 5620	64.6	0.14	Non-toxic
Conv	PHT Supreme Spray Oil	10951-16	Conv	0.017	Mineral oil	99	8042-47-5	3340	> 5620	64.6	0.14	Non-toxic
Conv	Prowl H2O Herbicide	241-418	Conv	0.017	Pendimethalin	38.7	40487-42-1	4665	1421	0.196	0.147	0.004
Conv	PHT Supreme Spray Oil	7001-7778	Conv	0.017	Mineral oil	99	64742-55-8	No record found	No record found	No record found	> 10,000	Non-toxic
Conv	Spray Oil 415	34704-727	Conv	0.016	Light and Heavy Paraffinic Distillates	98	64741-89-5, 64741-88-9, 64742-56-9, and 64742-65-0	> 5000	No record found	> 1000	> 1000	No record found
Conv	PHT Supreme Spray Oil	7001-7777	Conv	0.016	Mineral oil	99.7	8042-47-5	3340	> 5620	64.6	0.14	No record found
Conv	Wilbur-Ellis Dusting Sulfur	2935-48	Org	0.015	Sulfur	90-100	7704-34-9	> 2000	> 2000	> 0.063	> 0.063	> 0.063
Conv	First Choice Narrow Range 415 Spray Oil	34704-1025	Conv	0.015	Mineral oil	98	Mixture	> 5000	No record found	No record found	No record found	No record found

Conv	Rex Lime Sulfur Solution	71096-6	Org	0.014	Calcium Polysulfide	28	1344-81-6	1343	560	> 2.86	> 6.6	>12.6
Conv	Western lime - High Calcium Hydrated Lime	1051042-50001	Org	0.013	Calcium Hydroxide	90	1305-62-0	7340	No record found	33.9	No significant impact	79.22
Conv	Lime Sulfur Solution	61842-30	Org	0.011	Calcium Polysulfide	29	1344-81-6	1343	560	> 2.86	> 6.6	>12.6
Conv	Chemstar High Calcium Hydrated Lime	1057692-50001	Conv	0.011	Calcium Polysulfide	28	1344-81-6	1343	560	> 2.86	> 6.6	>12.6

Note: Ecological toxicity information for commonly used pesticides that contributed to ~50% of conventional and organic pesticide use in Kern County, CA from 2013 to 2019. Ecotoxicity records were matched to product CAS number(s) provided on the product Material Safety Data Sheet, product label, or to records of products with identical names if no CAS number was provided.

Supplementary Table 11. Pesticide metrics for organic and conventional fields

Pesticide Metric (Kg ha-1)	Management	Observations	Mean	SD
All Active Ingredients	Conventional	66,190	36.77	94.69
	Organic	3,989	18.32	36.45
All Products	Conventional	66,304	64.96	147.76
	Organic	3,993	38.94	61.87
Aquatic Species	Conventional	60,769	27.61	85.39
	Organic	3,506	22.74	39.25
Bees	Conventional	45,709	5.52	10.52
	Organic	2,813	7.32	11.89
Birds	Conventional	26,465	14.60	93.87
	Organic	366	4.62	7.09
Drift	Conventional	65,742	55.80	143.72
	Organic	3,648	30.91	55.85
Higher toxicity (EPA 1,2)	Conventional	60,731	35.70	130.23
	Organic	2,311	12.15	34.36
Lower toxicity (EPA 3,4)	Conventional	63,056	33.92	72.78
	Organic	3,854	33.06	47.54
Fish	Conventional	58,187	35.81	119.81
	Organic	3,234	18.78	38.12
Ground Water	Conventional	48,957	20.72	115.31
	Organic	4	0.72	1.02
Herbicides Only	Conventional	51,310	8.61	10.28
	Organic	128	25.59	22.01
Insecticides Only	Conventional	50,028	15.52	27.26
	Organic	2,852	9.28	19.33
Insect/Fungicide	Conventional	24,340	58.00	154.92
	Organic	2,761	25.63	47.82
Mammals	Conventional	25,973	34.39	146.90
	Organic	66	1.29	2.46
Reptiles, Amphibians	Conventional	8,454	1.46	1.13
	Organic	0		
Water	Conventional	66,187	61.48	146.65
	Organic	3,989	38.10	59.52
Wildlife	Conventional	45,246	5.09	12.10
	Organic	101	6.10	29.11

Note: Unless otherwise noted, metrics are kg ha^{-1} of pesticide product. Ecotoxicological outcomes (e.g. of potential concern to mammals, water, wildlife, etc.) and pesticide types (e.g. insecticides) based on the CDPR Product Database. Observations indicate the number of fields with non-zero pesticide use of a given designation. Mean and standard deviation are for fields with non-zero pesticide use of a given designation. Gray shading indicates which management type (organic, conventional) had a higher average use for a given pesticide metric.

Supplementary Table 12. Double hurdle models for 2017–2019 including self-reported organic status in addition to PUR Organic.

VARIABLES	(1)<br>All AI	(2)<br>All Prd	(3)<br>Drift	(5)<br>Insect.	(6)<br>Fish	(7)<br>Bee	(8)<br>EPA High	(9)<br>EPA Low
(A) Hurdle 1, binary								
Organic	-0.284**	-0.284**	-0.284**	-0.195**	-0.262**	-0.162**	-0.295**	-0.259**
	(0.032)	(0.032)	(0.032)	(0.024)	(0.027)	(0.022)	(0.035)	(0.030)
ln(Field Size)	0.028**	0.028**	0.027**	0.027**	0.028**	0.024**	0.026**	0.033**
	(0.004)	(0.004)	(0.004)	(0.006)	(0.005)	(0.004)	(0.004)	(0.006)
Soil Quality	-0.010**	-0.009**	-0.011**	-0.012**	-0.011**	-0.014**	-0.007*	-0.012**
	(0.003)	(0.003)	(0.003)	(0.004)	(0.004)	(0.005)	(0.003)	(0.003)
ln(Farm Size)	0.018**	0.019**	0.019**	0.003	0.013*	0.007	0.023**	0.016*
	(0.006)	(0.006)	(0.006)	(0.007)	(0.006)	(0.008)	(0.006)	(0.006)
Avg. Partial Effects	X	X	X	X	X	X	X	X
Farmer x family, Yr RE	X	X	X	X	X	X	X	X
Observations	40,002	40,002	40,002	40,002	40,002	40,002	40,002	40,002
(B) Hurdle 2, For Kg >0								
Organic	0.196	0.227	0.242	0.020	0.283	0.147	0.172	0.487**
	(0.184)	(0.169)	(0.180)	(0.178)	(0.229)	(0.195)	(0.249)	(0.176)
ln(Field Size)	-0.031	-0.057*	-0.055*	-0.040	-0.067**	-0.071**	-0.073*	-0.045*
	(0.032)	(0.023)	(0.024)	(0.027)	(0.025)	(0.026)	(0.032)	(0.020)
Soil Quality	-0.037*	-0.030*	-0.024~	-0.015	-0.045*	-0.012	-0.011	-0.028~
	(0.018)	(0.013)	(0.014)	(0.015)	(0.020)	(0.020)	(0.015)	(0.015)
ln(Farm Size)	-0.116~	-0.114*	-0.110*	-0.176**	-0.036	-0.090*	-0.128*	-0.137*
	(0.063)	(0.055)	(0.056)	(0.054)	(0.058)	(0.036)	(0.057)	(0.054)
Farmer x family	X	X	X	X	X	X	X	X
Observations	30,212	30,247	29,974	23,253	26,898	21,299	28,124	28,848

Notes: Lognormal double hurdle models estimating the effect of organic on the decision to spray (A) and the decision of how much to spray on fields using positive pesticide use (B) across different pesticide use metrics for 2017-2019, including as "organic" both observations self-reported as organic and fields we identified as PUR Organic (see text). Models include all active ingredients (AI), all products (Prd), Models include all active ingredients (AI), all products (Prd), chemicals functioning as insecticides only (Insect), chemicals prone to drift (Drift), chemicals of potential hazard to fish and bees (Fish, Bees), as well as chemicals with high and low acute toxicity based on the EPA signal word (High, Low). Abbreviations correspond to Supplementary Figure 2. We include farmer-by-crop family random effects. For all double hurdle models, we use the natural log of pesticide use metrics as the outcome and the natural log of field and farm size. In the first hurdle, which is estimated using a random effect probit, we report the average partial effects. Models include cluster robust errors clustered at farm-by-crop family with ~, *, ** indicating $p<0.1$, $p<0.05$, $p<0.01$, respectively, based on two-tailed t-test with $\alpha = 0.05$ and no adjustments for multiple comparisons.

Supplementary Table 13. Double hurdle models for 2017–2019 only, ignoring self-reported organic fields.

VARIABLES	(1)<br>All AI	(2)<br>All Prd	(3)<br>Drift	(5)<br>Insect.	(6)<br>Fish	(7)<br>Bees	(8)<br>EPA High	(9)<br>EPA Low
(A) Hurdle 1, binary								
Organic	-0.301**	-0.300**	-0.302**	-0.214**	-0.275**	-0.179**	-0.288**	-0.280**
	(0.034)	(0.034)	(0.033)	(0.026)	(0.029)	(0.024)	(0.038)	(0.032)
ln(Field Size)	0.029**	0.028**	0.028**	0.028**	0.029**	0.024**	0.027**	0.033**
	(0.004)	(0.004)	(0.004)	(0.006)	(0.005)	(0.004)	(0.004)	(0.006)
Soil Quality	-0.010**	-0.009**	-0.011**	-0.012**	-0.011**	-0.014**	-0.007*	-0.012**
	(0.003)	(0.003)	(0.003)	(0.004)	(0.004)	(0.005)	(0.003)	(0.003)
ln(Farm Size)	0.019**	0.019**	0.020**	0.004	0.014*	0.007	0.023**	0.016**
	(0.006)	(0.006)	(0.006)	(0.007)	(0.006)	(0.008)	(0.006)	(0.006)
Avg. Partial Effects	X	X	X	X	X	X	X	X
Farmer x family, Yr RE	X	X	X	X	X	X	X	X
Observations	40,002	40,002	40,002	40,002	40,002	40,002	40,002	40,002
(B) Hurdle 2, For Kg >0								
Organic	0.251	0.174	0.192	-0.068	0.259	-0.119	-0.041	0.390*
	(0.173)	(0.146)	(0.154)	(0.195)	(0.174)	(0.196)	(0.190)	(0.152)
ln(Field Size)	-0.032	-0.058*	-0.056*	-0.040	-0.068**	-0.073**	-0.074*	-0.047*
	(0.032)	(0.023)	(0.024)	(0.027)	(0.025)	(0.026)	(0.032)	(0.020)
Soil Quality	-0.037*	-0.030*	-0.024~	-0.015	-0.045*	-0.013	-0.011	-0.029*
	(0.018)	(0.013)	(0.014)	(0.015)	(0.020)	(0.020)	(0.015)	(0.015)
ln(Farm Size)	-0.117~	-0.113*	-0.109~	-0.175**	-0.037	-0.086*	-0.125*	-0.136*
	(0.063)	(0.055)	(0.056)	(0.055)	(0.058)	(0.037)	(0.057)	(0.054)
Farmer x family	X	X	X	X	X	X	X	X
Observations	30,212	30,247	29,974	23,253	26,898	21,299	28,124	28,848

Notes: Lognormal double hurdle models estimating the effect of organic on the decision to spray (A) and the decision of how much to spray on fields using positive pesticide use (B) across different pesticide use metrics for 2017-2019, including as "organic" only fields we identified as PUR Organic (see text). Models include all active ingredients (AI), all products (Prd), chemicals functioning as

insecticides only (Insect.), chemicals prone to drift (Drift), chemicals of potential hazard to fish and bees (Fish, Bees), as well as chemicals with high and low acute toxicity based on the EPA signal word (High, Low). Abbreviations correspond to Supplementary Figure 3. We include farmer-by-crop family random effects. We include farmer-by-crop family random effects. For all double hurdle models, we use the natural log of pesticide use metrics as the outcome and the natural log of field and farm size. In the first hurdle, which is estimated using a random effect probit, we report the average partial effects. Models include cluster robust errors clustered at farm-by-crop family with ~, *, ** indicating $p<0.1$, $p<0.05$, $p<0.01$, respectively, based on two-tailed t-test with $\alpha = 0.05$ and no adjustments for multiple comparisons.

Supplementary Table 14. Double hurdle models using kg of active ingredients rather than kg of products for different pesticide use outcomes.

VARIABLES	(1)<br>Insect.	(2)<br>Drift	(3)<br>Fish	(4)<br>Bee	(5)<br>High	(6)<br>Low
(A) Hurdle 1, binary						
Organic	-0.209**	-0.310**	-0.286**	-0.182**	-0.311**	-0.276**
	(0.022)	(0.028)	(0.025)	(0.021)	(0.025)	(0.025)
ln(Field Size)	0.024**	0.028**	0.030**	0.021**	0.033**	0.031**
	(0.004)	(0.004)	(0.003)	(0.003)	(0.003)	(0.004)
Soil Quality	-0.010**	-0.008**	-0.011**	-0.008**	-0.005~	-0.010**
	(0.003)	(0.002)	(0.003)	(0.003)	(0.003)	(0.003)
ln(Farm Size)	-0.003	0.021**	0.016**	-0.000	0.018**	0.017**
	(0.008)	(0.005)	(0.005)	(0.008)	(0.005)	(0.006)
Avg. Partial Effects	X	X	X	X	X	X
Farmer x family, Yr RE	X	X	X	X	X	X
Observations	91,926	91,926	91,926	91,926	91,926	91,926
(B) Hurdle 2, For Kg >0						
Organic	-0.380~	-0.099	-0.028	-0.227	0.020	0.195
	(0.210)	(0.140)	(0.187)	(0.231)	(0.171)	(0.122)
ln(Field Size)	0.016	0.008	-0.022	-0.018	-0.029	-0.004
	(0.024)	(0.019)	(0.023)	(0.021)	(0.029)	(0.019)
Soil Quality	-0.022	-0.018	-0.048**	-0.019	-0.007	-0.019
	(0.019)	(0.012)	(0.015)	(0.020)	(0.015)	(0.015)
ln(Farm Size)	-0.161**	-0.108*	-0.047	-0.071*	-0.027	-0.128*
	(0.042)	(0.050)	(0.049)	(0.031)	(0.056)	(0.055)
Farmer x family	X	X	X	X	X	X
Observations	52,606	67,967	60,638	48,254	55,106	64,114

Notes: Lognormal double hurdle models estimating the effect of organic on the decision to spray (A) and the decision of how much to spray on fields using positive pesticide use (B) across different pesticide use metrics based on active ingredients: chemicals functioning as insecticides only (Insect.), chemicals prone to drift (Drift), chemicals of potential hazard to fish and bees (Fish, Bees), as well as chemicals with high and low acute toxicity based on the EPA signal word (High, Low). Abbreviations correspond to Supplementary Figure 4. We include farmer-by-crop family random effects. For all double hurdle models, we use the natural log of pesticide use metrics as the outcome and the natural log of field and farm size (in contrast to ihs transformation in table 1, see text). In the first hurdle, which is estimated using a random effect probit, we report the average partial effects. Models include cluster robust errors clustered at farm-by-crop family with ~, *, ** indicating $p<0.1$, $p<0.05$, $p<0.01$, respectively, based on two-tailed t-test with $\alpha = 0.05$ and no adjustments for multiple comparisons.

Supplementary References

1. Ponisio, L. C. *et al.* Diversification practices reduce organic to conventional yield gap. *Proc. R. Soc. B Biol. Sci.* **282**, 20141396 (2015).
