# Supplementary material for: Identifying and characterizing pesticide use on 9,000 fields of organic agriculture
Source: Nat Commun. 2021 Sep 15;12:5461. doi: 10.1038/s41467-021-25502-w (PMC8443594; doi:10.1038/s41467-021-25502-w)
Supplement: Supplementary file 5 — Reporting Summary [file 41467_2021_25502_MOESM5_ESM.pdf]

nature research

Corresponding author(s): Ashley E Larsen

Last updated by author(s): Jul 26, 2021

Reporting Summary

Nature Research wishes to improve the reproducibility of the work that we publish. This form provides structure for consistency and transparency in reporting. For further information on Nature Research policies, see our Editorial Policies and the Editorial Policy Checklist.

Statistics

For all statistical analyses, confirm that the following items are present in the figure legend, table legend, main text, or Methods section.

n/a	Confirmed	
☐	☒	The exact sample size (*n*) for each experimental group/condition, given as a discrete number and unit of measurement
☒	☐	A statement on whether measurements were taken from distinct samples or whether the same sample was measured repeatedly
☐	☒	The statistical test(s) used AND whether they are one- or two-sided<br>*Only common tests should be described solely by name; describe more complex techniques in the Methods section.*
☐	☒	A description of all covariates tested
☐	☒	A description of any assumptions or corrections, such as tests of normality and adjustment for multiple comparisons
☐	☒	A full description of the statistical parameters including central tendency (e.g. means) or other basic estimates (e.g. regression coefficient) AND variation (e.g. standard deviation) or associated estimates of uncertainty (e.g. confidence intervals)
☐	☒	For null hypothesis testing, the test statistic (e.g. *F*, *t*, *r*) with confidence intervals, effect sizes, degrees of freedom and *P* value noted<br>*Give P values as exact values whenever suitable.*
☒	☐	For Bayesian analysis, information on the choice of priors and Markov chain Monte Carlo settings
☐	☒	For hierarchical and complex designs, identification of the appropriate level for tests and full reporting of outcomes
☐	☒	Estimates of effect sizes (e.g. Cohen's *d*, Pearson's *r*), indicating how they were calculated

Our web collection on statistics for biologists contains articles on many of the points above.

Software and code

Policy information about availability of computer code

Data collection	We identified organic fields using a combination of California Department of Food and Agriculture (CDFA) records and Kern County Agricultural Commissioner's Office spatial data ("fields shapefiles") and pesticide use records.<br>All spatial data collection were performed in R Statistical Software v 3.5.3. R packages used for data collection: tidyverse (v1.3.0),reshape2 (v1.4.4), rgdal (v1.5.8), sf (v0.9.3),gstat (v2.0.7), raster (v3.1.5), sp (v1.4.2), fasterize (v1.0.2), rgdal (v1.5.8), cleangeo (v0.2.3), rgeos (v0.5.3), haven (v2.3.1),PesticideLoadIndicator (v0.0.1),webchem (v1.1.1),standartox (v0.0.1),data.table (v1.14.0).
Data analysis	All statistical analyses were performed Stata 16 MP. Code to repeat the main analysis is available in Supplementary Data File 2.<br>Stata commands used for data analysis & visualization: meprobit, mixed, coefplot, eststo, outreg2, reg, reghdfe, nlcom.

For manuscripts utilizing custom algorithms or software that are central to the research but not yet described in published literature, software must be made available to editors and reviewers. We strongly encourage code deposition in a community repository (e.g. GitHub). See the Nature Research guidelines for submitting code & software for further information.

Data

Policy information about availability of data

All manuscripts must include a data availability statement. This statement should provide the following information, where applicable:

- Accession codes, unique identifiers, or web links for publicly available datasets
- A list of figures that have associated raw data
- A description of any restrictions on data availability

Agriculture and pesticide use data for Kern County are available at http://www.kernag.com/gis/gis-data.asp. The California Department of Pesticide Regulation Product Database is available at https://apps.cdpr.ca.gov/docs/label/labelque.cfm and Kern County Parcel data is available at https://geodat-

kernco.opendata.arcgis.com/. Soil quality data are from Natural Resource Conservation Service SSUGO data available from https://websoilsurvey.nrcs.usda.gov/ and accessed using https://websoilsurvey.sc.egov.usda.gov/App/WebSoilSurvey.aspx . Data on registered organic producers was obtained through request from the California State Organics Program, https://www.cdfa.ca.gov/is/organicprogram/. Hand classified organic and conventional pesticides are provided in Supplementary Data File 1. Data to repeat the main analyses are available on Dryad, https://doi.org/10.25349/D9Q02T. Regression tables underlying Figures 2–4 are available in the Supplementary Information and source code for all analysis figures (main text, supplementary) are available in the Source Code zipfile.

Field-specific reporting

Please select the one below that is the best fit for your research. If you are not sure, read the appropriate sections before making your selection.

☐ Life sciences ☐ Behavioural & social sciences ☒ Ecological, evolutionary & environmental sciences

For a reference copy of the document with all sections, see nature.com/documents/nr-reporting-summary-flat.pdf

Ecological, evolutionary & environmental sciences study design

All studies must disclose on these points even when the disclosure is negative.

Study description	Our goal is to quantify the differences in total pesticide use and pesticides of specific concern to different ecological and environmental end points to further understanding of the environmental benefits and drawbacks of different production systems. We harmonize and aggregate several data sources to identify the spatial location of organic crop fields, and rely on unique, field-level crop and pesticide use data from Kern County, California to understand pesticide use difference.<br>Our investigation primarily relies on double hurdle models to parse apart the decision to spray pesticides from the decision of how much to spray. Using these models, we evaluate (1) overall differences between organic and conventional fields with respect to the decisions to spray and how much to spray for total pesticide use and pesticides of potential hazard to a range of different endpoints, (2) crop-specific differences in pesticide use decisions between organic and conventional fields for five crops commonly grown with both organic and conventional practices, and (3) how adjusting for yield gaps may influence the overall results.<br><br>The statistical approach is further described in "Randomization" below.<br>To assess the potential implications of a yield gap on our results, we modify our per hectare pesticide use rates following Ponisio et al. 2015 (https://doi.org/10.1098/rspb.2014.1396) as a robustness check. We group commodities into cereals, roots and tubers, oilseeds, legumes/pulses, fruits, and vegetables and assign them the Ponisio et al. (2015) yield gap estimates for that group. Crops that did not fall into any of the above groups were (e.g., cannabis) provided the all crop average from Ponisio et al. (2015). Lastly, we analyze how conventional and organic differ with respect to soil quality using a within estimator approach to account for crop-specific differences in soil quality.
Research sample	Our full sample consisted of 99,533 fields (organic and conventional fields combined). This represents all fields in Kern County, CA based on the Kern County Agricultural Commissioner's spatial data (available on http://www.kernag.com/gis/gis-data.asp) between 2013-2019.
Sampling strategy	Please see "Research sample" above. The start and end dates (2013-2019) were defined by the availability of CDFA records of organic agriculture.
Data collection	This study used secondary data from California Department of Food and Agriculture (CDFA) and Kern County Agricultural Commissioner's Office spatial data ("fields shapefiles") and pesticide use records.
Timing and spatial scale	All fields in Kern County, CA based on the Kern County Agricultural Commissioner's spatial data (available on http://www.kernag.com/gis/gis-data.asp) between 2013-2019.
Data exclusions	Observations with ambiguous crop family were dropped in any models including family in either the random effects or the cluster robust standard errors. While 7,367 fields were dropped due to missing crop family, 6,684 of those were uncultivated agriculture. A small number of observations (n = 319) were dropped due to missing soil quality data. Including observations with interpolated soil quality has little effect on our results.
Reproducibility	Code to reproduce the main results is available in the supplementary data. Data to repeat the main analysis is available on Dryad. This study used exclusively secondary data collected by county and state agencies.<br><br>Additional information on the statistical approach and methods considered is described in the randomization section below.
Randomization	Our statistical analysis proceeded in two steps. First, we evaluated whether conventional versus organic fields differed in pesticide use, modeled as a continuous variable, using pooled ordinary least squares and panel data models to determine the influence of different model specification decisions (see SI text, SI table 2-3). However, pesticide use can be conceived as a two-part decision. First, there is the decision to use pesticides at all, and second is the decision of how much to spray when using pesticides. Tobit models are traditionally used to estimate models with censoring. However, Tobit models force the mechanisms determining whether to spray (i.e., moving from pesticide = 0 to pesticides > 0) to be the same as the mechanisms determining the amount sprayed when some pesticides are used (pesticides when pesticides > 0). Double-hurdle models are an alternative to the Tobit model that allow for the separation of these two decisions.<br>For the first hurdle, we are interpreting the use of zero pesticides as the true choice of the farmer and are predicting the probability the farmer (of a given field) is "zero type" as a function of being organic or not. We do so using a random effects probit model with covariates for field size, farm size, and soil quality, with random intercepts for farm-by-crop family and with cluster robust standard errors clustered at the farm-by-crop family (SI text). In the second hurdle, we evaluate what drives the amount of pesticide use on

fields that decide to spray using a linear model with pesticide use modeled as lognormally distributed, conditional on covariates and for positive pesticide use observations. We use a lognormal hurdle model rather than a truncated normal hurdle model since pesticide use is highly non-normal, and Q-Q plots suggested substantial model improvement using a lognormal rather than normal distribution.

Blinding: Blinding was not relevant for this study. This study used secondary data exclusively.

Did the study involve field work? ☐ Yes ☒ No

Reporting for specific materials, systems and methods

We require information from authors about some types of materials, experimental systems and methods used in many studies. Here, indicate whether each material, system or method listed is relevant to your study. If you are not sure if a list item applies to your research, read the appropriate section before selecting a response.

Materials & experimental systems

n/a	Involved in the study
☒	☐ Antibodies
☒	☐ Eukaryotic cell lines
☒	☐ Palaeontology and archaeology
☒	☐ Animals and other organisms
☒	☐ Human research participants
☒	☐ Clinical data
☒	☐ Dual use research of concern

Methods

n/a	Involved in the study
☒	☐ ChIP-seq
☒	☐ Flow cytometry
☒	☐ MRI-based neuroimaging
