# Supplementary material for: Identifying and characterizing pesticide use on 9,000 fields of organic agriculture
Source: Nat Commun. 2021 Sep 15;12:5461. doi: 10.1038/s41467-021-25502-w (PMC8443594; doi:10.1038/s41467-021-25502-w)
Supplement: Supplementary file 6 — Description of Additional Supplementary Files [file 41467_2021_25502_MOESM6_ESM.docx]

Description of Additional Supplementary Files

File Name: Supplementary Data 1

Description: Pesticides determined to be organic acceptable (or not) based on review of pesticide labels and Organic Materials Review Institute certification database. Please see description in the methods (“Using Pesticide Use Reports to Refine Organic Field Identification”).

File Name: Supplementary Data 2

Description: Stata code to repeat the main analyses.
